# Heightened JNK Activation and Reduced XIAP Levels Promote TRAIL and Sunitinib-Mediated Apoptosis in Colon Cancer Models

**DOI:** 10.3390/cancers11070895

**Published:** 2019-06-26

**Authors:** Devalingam Mahalingam, Jennifer S. Carew, Claudia M. Espitia, Robbert H. Cool, Francis J. Giles, Steven de Jong, Steffan T. Nawrocki

**Affiliations:** 1 Department of Medicine, Robert H. Lurie Comprehensive Cancer Center, Northwestern University, Chicago, IL 60611, USA; 2 Department of Medicine, University of Arizona Cancer Center, Tucson, AZ 85724, USA; 3 Department of Chemical and Pharmaceutical Biology, Groningen Research Institute of Pharmacy (GRIP), University of Groningen, 9713 GZ Groningen, The Netherlands; 4 Department of Medical Oncology, University Medical Centre Groningen, University of Groningen, 9713 GZ Groningen, The Netherlands

**Keywords:** TRAIL, Apoptosis, sunitinib, XIAP, colon cancer

## Abstract

Tumor necrosis factor-related apoptosis-inducing ligand (TRAIL) is a potent inducer of apoptosis that may be a promising agent in cancer therapy due to its selectivity toward tumor cells. However, many cancer cells are resistant to TRAIL due to defects in apoptosis signaling or activation of survival pathways. We hypothesized that a disruption of pro-survival signaling cascades with the multi-tyrosine kinase inhibitor sunitinib would be an effective strategy to enhance TRAIL-mediated apoptosis. Here we demonstrate that sunitinib significantly augments the anticancer activity of TRAIL in models of colon cancer. The therapeutic benefit of the TRAIL/sunitinib combination was associated with increased apoptosis marked by enhanced caspase-3 cleavage and DNA fragmentation. Overexpression of the anti-apoptotic factor B-cell lymphoma 2 (BCL-2) in HCT116 cells reduced TRAIL/sunitinib-mediated apoptosis, further supporting that sunitinib enhances the anticancer activity of TRAIL via augmented apoptosis. Analysis of pro-survival factors identified that the combination of TRAIL and sunitinib significantly downregulated the anti-apoptotic protein X-linked inhibitor of apoptosis protein (XIAP) through a c-Jun N-terminal kinase (JNK)-mediated mechanism. Short hairpin RNA (shRNA)-mediated knockdown of JNK confirmed its key role in the regulation of sensitivity to this combination as cells with suppressed JNK expression exhibited significantly reduced TRAIL/sunitinib-mediated apoptosis. Importantly, the therapeutic benefit of the TRAIL/sunitinib combination was validated in the HCT116-Luc and HCT15 colon cancer xenograft models, which both demonstrated significant anti-tumor activity in response to combination treatment. Collectively, our data demonstrate that sunitinib enhances TRAIL-mediated apoptosis by heightened JNK activation, diminished XIAP levels, and augmented apoptosis.

## 1. Introduction

The tumor necrosis factor (TNF) superfamily member tumor necrosis factor-related apoptosis-inducing ligand (TRAIL) is a potent inducer of apoptosis that may have significant broad ranging applications in cancer therapy due to its selectivity toward cancer cells [1]. TRAIL induces the extrinsic apoptotic pathway upon binding to its death domain (DD)-containing receptors, TRAIL receptor 1 (death receptor 4; DR4) and 2 (death receptor 5; DR5). Binding of TRAIL to DR4 and DR5 induces receptor oligomerization, intracellular DD clustering and recruitment of the adaptor molecule Fas associated death domain (FADD). The death effector domains (DED) of FADD interact with the DED of pro-caspase-8/-10 leading to the formation of the death inducing signal complex (DISC). The DISC serves as a platform to oligomerize and activate pro-caspase-8/-10 [2,3]. Active caspase-8/-10 is released from the DISC, which then activates the executioner caspases-3 -6 and -7 committing the cell to death.

Despite the significant anticancer activity of TRAIL against many cancer cells, its potential clinical impact as an anticancer agent could be tempered by endogenous resistance, which may occur due to defects in apoptosis signaling or activation of survival pathways. For example, upregulation of B-cell lymphoma 2 (BCL-2) family proteins may elicit resistance to TRAIL-mediated apoptosis [4,5,6]. Another potent blocker of apoptosis, inhibitor of apoptosis proteins (IAP), are highly expressed in some types of cancer cells and have been shown to diminish TRAIL-mediated apoptosis [7]. Among IAP family members, X-linked inhibitor of apoptosis protein (XIAP) is considered to be the most potent direct inhibitor of caspase activity and has become a strategic target to sensitize cancer cells to apoptosis stimulated by a number of regimens [8,9].

c-Jun N-terminal kinase (JNK) can activate a number of diverse downstream targets including members of the activator protein-1 (AP-1) family, c-JUN, BCL-2 proteins, c-MYC, and p53 depending on the cell type and the specific stimulus, [10,11]. We previously showed the JNK is activated by TRAIL in colon cancer cells. Our collective findings demonstrated that this event plays an important role in TRAIL-induced cell death in this cancer type [12]. Therefore, precision therapeutic strategies that promote heightened JNK activation may augment TRAIL-mediated apoptosis in colon cancer and other malignancies that exhibit sensitivity to TRAIL [13,14].

Sunitinib is a selective multi-targeted receptor tyrosine kinase (RTK) inhibitor that exhibits direct anticancer activity against tumor cells through the blockade of vascular endothelial growth factor (VEGF) receptor (R)-1, 2 and 3, platelet-derived growth factor receptor (PDGFR)-ɑ and -β, FLT3, RET, and KIT signaling [15]. Consistent with this mechanism of action, inhibition of angiogenesis has been validated as the key aspect of sunitinib’s antitumor mechanism of action [16,17,18]. In addition to its prominent effects on the tumor vascular network, sunitinib also exhibits direct activity against cancer cells by stimulating apoptosis. Rational synergistic interactions between TRAIL and various RTK inhibitors have been previously investigated and have yielded an enhancement in TRAIL-mediated apoptosis [19,20,21]. We hypothesized that targeted disruption of pro-survival signaling cascades with sunitinib will strongly enhance TRAIL-mediated apoptosis. Here we demonstrate that sunitinib significantly augments TRAIL-induced JNK phosphorylation, which leads to downregulation of XIAP and enhanced anticancer activity in both in vitro and in vivo colon cancer models. Our study provides the rationale of targeting XIAP with sunitinib to enhance TRAIL-mediated apoptosis in cancer cells where XIAP plays a pivotal role in driving apoptotic resistance.

## 2. Results

### 2.1. Sunitinib Potentiates the Pro-Apoptotic Activity of Recombinant Human (rh)TRAIL in Colon Cancer Cell Models

**Figure 1:**
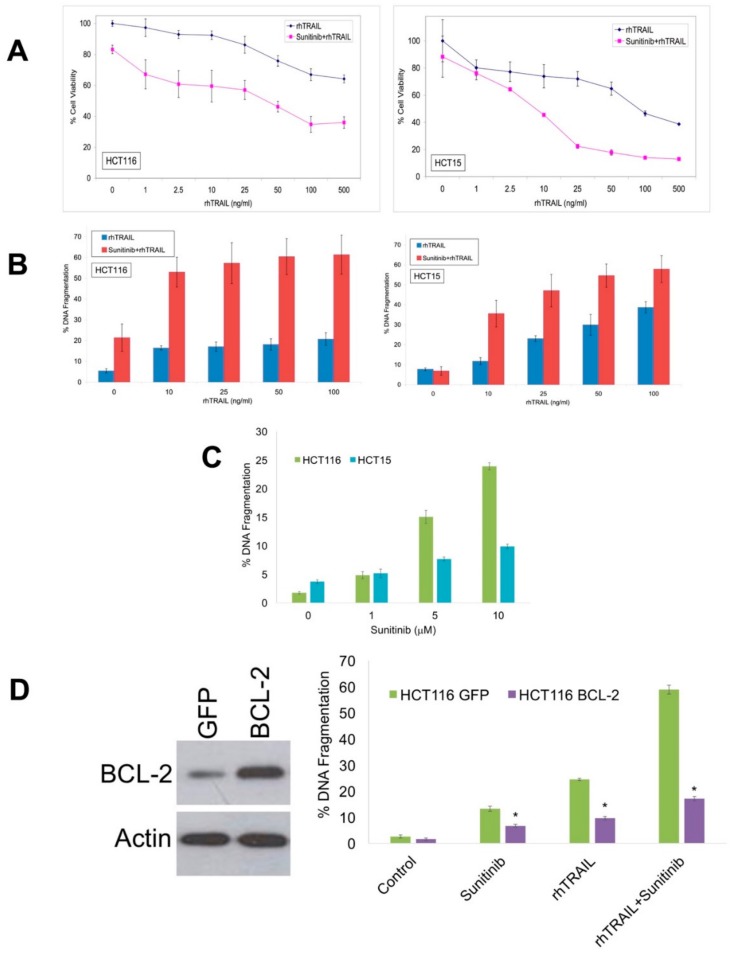
Sunitinib enhances the activity of recombinant human tumor necrosis factor-related apoptosis-inducing ligand (rhTRAIL) in colon cancer cell lines. (**A**) Cells were treated for 72 h with 5 μM sunitinib and/or the indicated concentrations of rhTRAIL. Viability was assessed by MTT assay. (**B**) Sunitinib promotes rhTRAIL-mediated apoptosis. Cells were treated with 5 μM sunitinib and the indicated concentrations of rhTRAIL for 24 h. Apoptosis (DNA fragmentation) was determined by propidium iodide staining followed by flow cytometric analysis. (**C**) Sunitinib induces apoptosis in colon cancer cells. HCT116 and HCT15 were treated with the indicated concentrations of sunitinib for 24 h. Apoptosis was measured by propidium iodide fluorescence activated cell sorting (PI-FACS) analysis. Mean ± Standard Deviation (SD), *n* = 3. (**D**) HCT116 cells were stably transfected with control vector (GFP) or B-cell lymphoma 2 (BCL-2). Overexpression of BCL-2 was confirmed by immunoblotting. Cells were treated with 5 μM sunitinib, 100 ng/L rhTRAIL, or the combination for 24 h. Apoptosis was determined by propidium iodide staining followed by flow cytometric analysis. Mean ± Standard Deviation, *n* = 3. * Indicates a significant difference compared to HCT116 GFP cells, *p* < 0.05.

To examine the sensitivity of colon cancer cells to TRAIL, HCT116, and HCT15 cells were treated with increasing concentrations of rhTRAIL alone or in combination with sunitinib for 24 h and cell viability was assessed by 3-(4,5-dimethylthiazol-2-yl)-2,5-diphenyltetrazolium bromide (MTT) assay (Figure 1A). rhTRAIL decreased cell viability in a dose-dependent manner. Sunitinib significantly enhanced the anticancer activity of rhTRAIL at multiple concentrations (Figure 1A). Propidium iodide (PI) staining and fluorescence activated cell sorting (FACS) analysis of both HCT116 and HCT15 colon cancer cell lines revealed that sunitinib significantly augments rhTRAIL-mediated apoptosis (Figure 1B). Treatment with sunitinib as a single agent induced only modest levels of apoptosis by PI-FACS analysis (Figure 1C). This is consistent with prior reports that sunitinib displays much less efficacy in vitro compared to in vivo as this assay does not account for the potent angiogenesis inhibition following sunitinib therapy [18,22]. To assess if enhanced apoptotic cell death contributes to the anticancer activity of the rhTRAIL and sunitinib combination, HCT116 cells were stably transfected with either control GFP vector or BCL-2. Overexpression of BCL-2 was confirmed by immunoblotting (Figure 1D). Further analysis by PI-FACS analysis demonstrated that overexpression of BCL-2 significantly reduced apoptosis induced by rhTRAIL, sunitinib, or the combination (Figure 1D). Collectively, the data demonstrate that enhanced apoptosis significantly contributes to the anticancer activity of the rhTRAIL and sunitinib combination.

### 2.2. JNK Activation Is a Critical Mediator of Apoptosis Following Treatment with rhTRAIL and Sunitinib 

**Figure 2:**
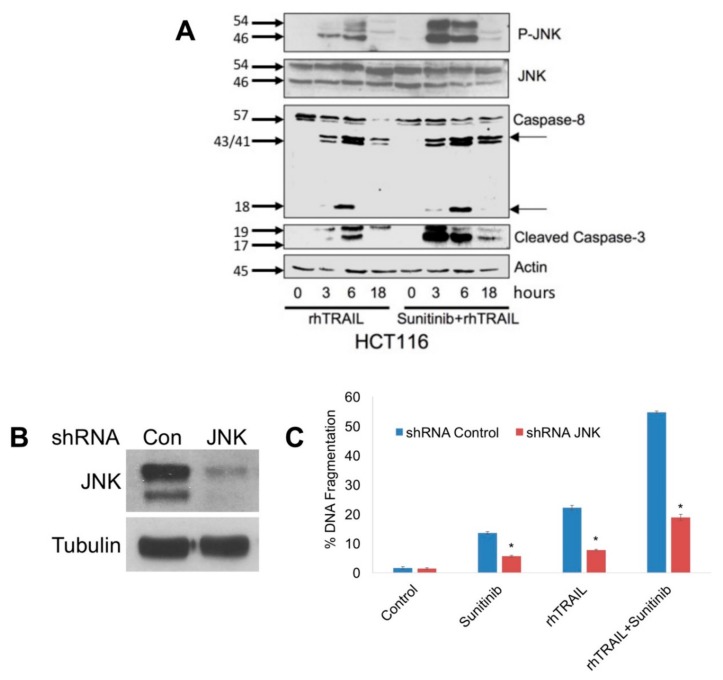
Sunitinib enhances the recombinant human tumor necrosis factor-related apoptosis-inducing ligand (rhTRAIL)-mediated c-Jun N-terminal kinase (JNK) phosphorylation, which significantly contributes to apoptosis induced by rhTRAIL, sunitinib, and the combination. (**A**) Sunitinib enhances rhTRAIL-mediated JNK phosphorylation and caspase-3 cleavage. HCT116 cells were treated with 100 ng/mL rhTRAIL, 5 μM sunitinib, or the combination for the indicated times (hours). JNK phosphorylation, caspase-8, and caspase-3 levels were measured by immunoblotting. (**B**) Knockdown of JNK levels. JNK expression was silenced using JNK-targeted short hairpin RNA (shRNA). Protein knockdown was evaluated by immunoblotting. (**C**) Knockdown of JNK reduces apoptosis induced by rhTRAIL, sunitinib, and the combination. Cells were treated with 100 ng/mL rhTRAIL, 5 μM sunitinib and the combination for 24 h. Apoptosis was determined by propidium iodide fluorescence activated cell sorting (PI-FACS) analysis. Mean ± Standard Deviation (SD), *n* = 3. * Indicates a significant difference compared to shRNA Control cells, *p* < 0.05.

Activation of JNK has been previously reported to contribute to TRAIL-mediated apoptosis [23,24,25]. To investigate whether the JNK pathway may control TRAIL-induced apoptosis of colon cancer cells, phosphorylation of JNK was first assessed by immunoblotting following treatment with rhTRAIL, sunitinib, and the combination. Treatment with 100 ng/mL of rhTRAIL resulted in phosphorylation of JNK in HCT116 cells. Phosphorylation of JNK was observed as early as 3-hour post-treatment with rhTRAIL. While sunitinib did not exhibit a major effect on JNK phosphorylation, co-treatment with rhTRAIL resulted in significantly higher levels of JNK phosphorylation than either single agent yielded. Enhanced JNK phosphorylation was associated with enhanced caspase cleavage (Figure 2A). To determine if JNK is an essential regulator of TRAIL-induced apoptosis, lentiviral shRNA was utilized to knockdown JNK expression in HCT116 cells (Figure 2B). DNA fragmentation analysis demonstrated that genetic diminishment of JNK levels significantly decreased apoptosis stimulated by rhTRAIL, sunitinib, and the combination (Figure 2C). Collectively, these data demonstrate that sunitinib improved TRAIL-induced apoptosis through enhanced JNK activation and that this may be a clinically actionable strategy to improve the anticancer activity of TRAIL in colon cancers.

### 2.3. Diminished XIAP Expression Drives the Pro-Apoptotic Effects of the rhTRAIL and Sunitinib Combination

**Figure 3:**
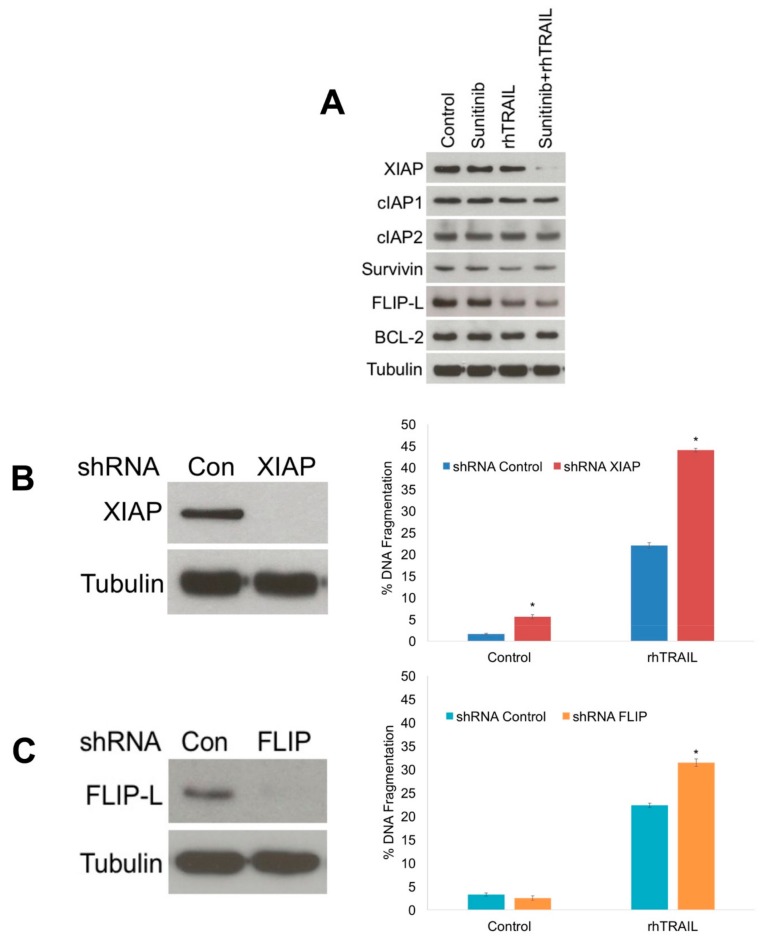
X-linked inhibitor of apoptosis protein (XIAP) downregulation sensitizes HCT116 cells to recombinant human tumor necrosis factor-related apoptosis-inducing ligand (rhTRAIL)-mediated apoptosis. (**A**) HCT116 cells were treated with rhTRAIL, sunitinib, and the combination for 24 h. IAPs, FLIP-L, and BCL-2 levels were measured by immunoblotting. (**B**,**C**) Knockdown of XIAP and FLIP sensitizes HCT116 cells to rhTRAIL-mediated apoptosis. XIAP and FLIP expression were silenced by shRNA. Cells were treated with 100 ng/mL rhTRAIL for 24 h and apoptosis was measured by propidium iodide fluorescence activated cell sorting (PI-FACS) analysis. Mean ± Standard Deviation (SD), *n* = 3. * Indicates a significant difference compared to shRNA Control cells, *p* < 0.05.

**Figure 4:**
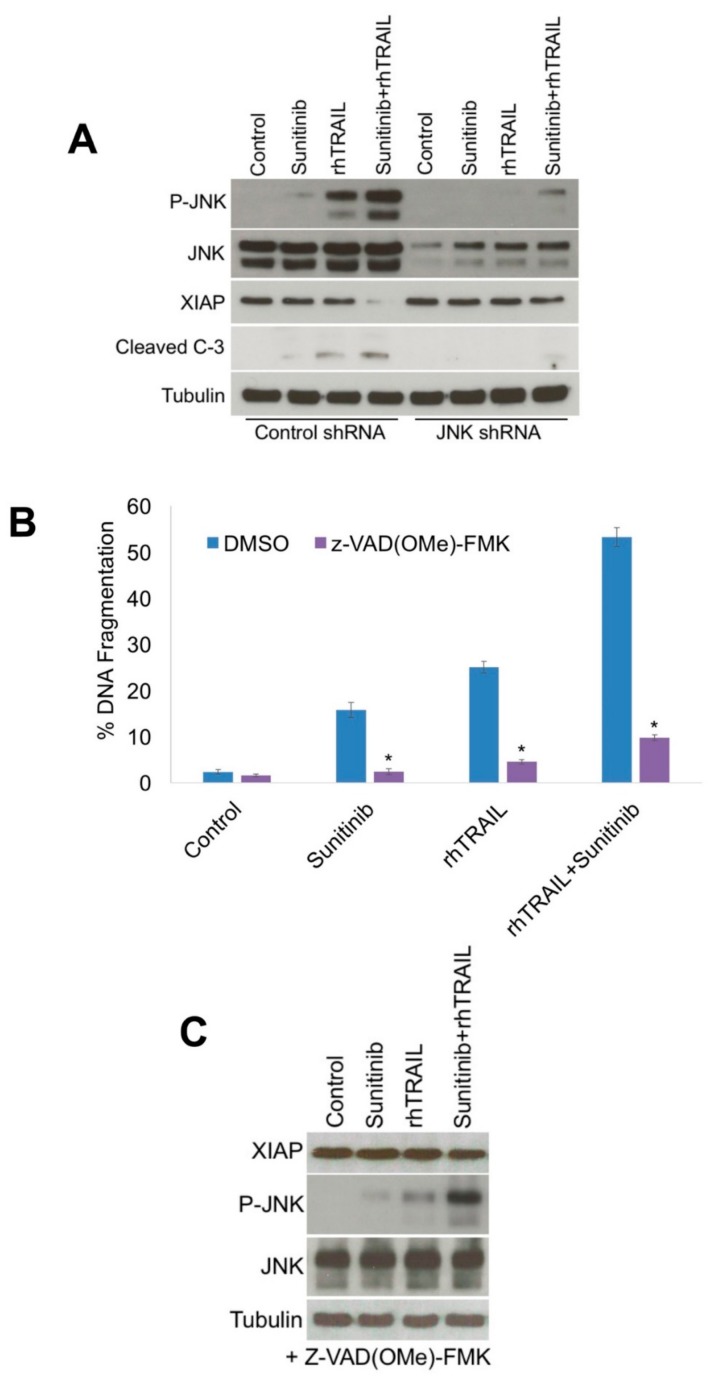
c-Jun N-terminal kinase (JNK) phosphorylation and caspase activation contribute to reduction of X-linked inhibitor of apoptosis protein (XIAP) levels. (**A**) Cells transfected with Control or JNK shRNA were treated with 100 ng/mL recombinant human tumor necrosis factor-related apoptosis-inducing ligand (rhTRAIL), 5 μM sunitinib, and the combination for 24 h. XIAP, p-JNK, JNK, and cleaved caspase-3 levels were determined by immunoblotting. (**B**) Treatment with the caspase inhibitor Z-VAD(OMe)-FMK inhibits apoptosis induced by rhTRAIL, sunitinib, and the combination. HCT116 cells were treated with 100 ng/mL rhTRAIL, 5 μM sunitinib, or the combination for 24 h with or without 10 μM Z-VAD(OMe)-FMK and apoptosis was measured by propidium iodide fluorescence activated cell sorting (PI-FACS) analysis. Mean ± Standard Deviation (SD), *n* = 3. * Indicates a significant difference compared to Control cells, *p* < 0.05. (**C**) Z-VAD(OMe)-FMK inhibits XIAP reduction induced by rhTRAIL and sunitinib combination treatment. HCT116 cells were treated with 100 ng/mL rhTRAIL, 5 μM sunitinib, or the combination for 24 h with or without 10 μM Z-VAD(OMe)-FMK and XIAP, p-JNK, and JNK levels were determined by immunoblotting.

Interestingly, the enhanced JNK phosphorylation stimulated by the rhTRAIL/sunitinib combination was correlated with enhanced cleavage of caspase-3 and a more limited effect on caspase-8 activation (Figure 2A). This result suggested that sunitinib could be antagonizing or diminishing factors such as the IAPs that function to inhibit apoptosis through regulation of caspase-3 [26]. To further investigate this possibility, we measured the effects of rhTRAIL, sunitinib, and the combination on the key anti-apoptotic factors IAPs, FLICE-like inhibitory protein (FLIP), and BCL-2 by immunoblotting. The combination induced a dramatic decrease in XIAP, a modest decrease in FLIP, and only minor alterations in the other proteins evaluated (Figure 3A). To further investigate the roles of FLIP and XIAP to rhTRAIL-mediated apoptosis, lentiviral shRNA was utilized to specifically silence FLIP and XIAP expression (Figure 3B,C). Measurement of apoptosis by PI-FACS analysis demonstrated that depleting both FLIP and XIAP levels significantly enhanced rhTRAIL-induced apoptosis (Figure 3B,C). However, silencing of XIAP resulted in a more dramatic enhancement in rhTRAIL-mediated apoptosis compared with FLIP. Therefore, we focused on further evaluation of the role of XIAP in rhTRAIL and sunitinib-induced cell death. JNK has been previously reported to enhance the cleavage of XIAP as one of its pro-apoptotic mechanisms of action [8,27]. To evaluate the link between JNK and XIAP, we measured XIAP and cleaved caspase-3 levels following knockdown of JNK expression (Figure 4A). Consistent with JNK activation playing a key role in rhTRAIL/sunitinib-mediated apoptosis, XIAP levels were not dramatically reduced and caspase-3 cleavage was inhibited following combination treatment in the absence of high JNK phosphorylation (Figure 4A). These data suggest that heightened JNK activation following rhTRAIL/sunitinib treatment results in downregulation of XIAP, which promotes sensitization to apoptosis. To evaluate the contribution of caspase activation to rhTRAIL and sunitinib induced apoptosis, cells were co-treated with the pan-caspase inhibitor z-vad(OMe)-fmk. Blocking caspase activation significantly decreased apoptosis induced by both single agents and the combination (Figure 4B). In addition, z-vad(OMe)-fmk also inhibited the reduction in XIAP expression, but not JNK phosphorylation stimulated by rhTRAIL and sunitinib combination treatment (Figure 4C). These results demonstrate that caspase activation is important for XIAP reduction and apoptosis mediated by the rhTRAIL and sunitinib combination.

### 2.4. Sunitinib and rhTRAIL Reduce Tumor Burden in Colon Cancer Xenografts

**Figure 5:**
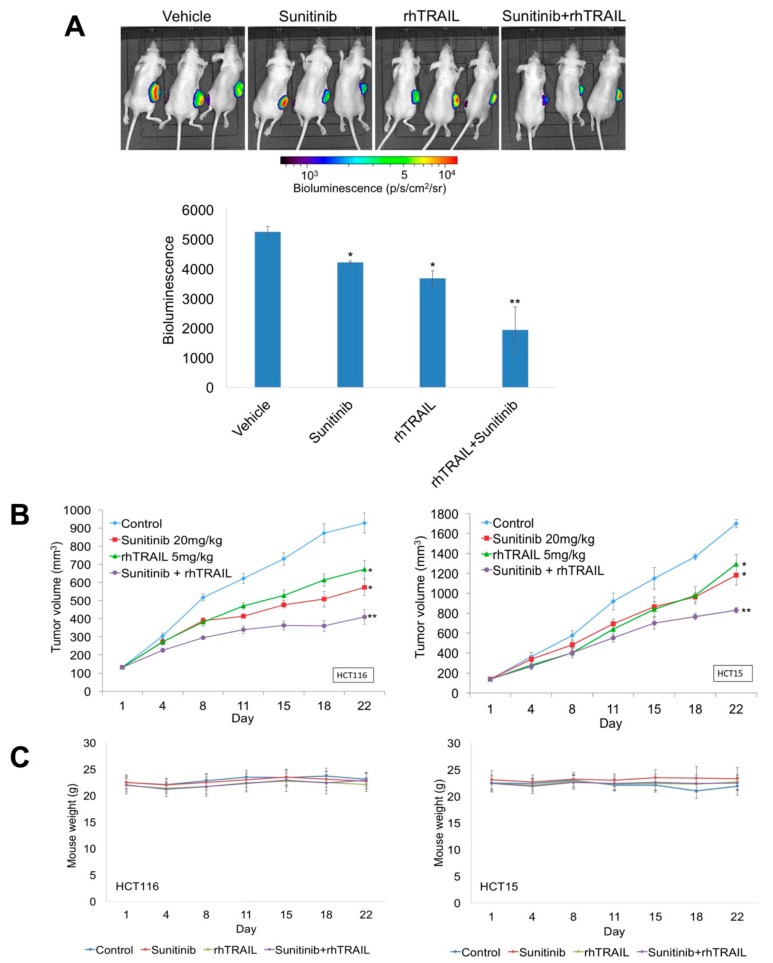
Sunitinib and recombinant human tumor necrosis factor-related apoptosis-inducing ligand (rhTRAIL) reduce tumor burden in colon cancer xenografts. (**A**,**B**) HCT116-luc and HCT15 colon cancer cells were injected s.c. into the flanks of nude mice. Mice with palpable tumors were randomized into groups of 10 and were treated with 20 mg/kg sunitinib orally (PO), 5 mg/kg rhTRAIL intraperitoneal (IP), or both agents daily for 5 days a week for 3 weeks. HCT116-luc tumor-bearing mice were imaged on Day 22. Tumor volume was measured twice weekly throughout the study. Mean ± standard error of the mean (SEM), *n* = 10. * Indicates a significant difference compared to vehicle control or ** compared to either single agent group, *p* < 0.05. (**C**) Drug treatments were well tolerated. Mouse weights were measured twice per week. Mean ± standard deviation (SD), *n* = 10.

To rigorously validate the potential benefit of rhTRAIL in combination with sunitinib, two separate xenograft studies were performed. Luciferase expressing HCT116 (HCT116-luc) and HCT15 cells were implanted into nude mice. Tumor-bearing animals were randomized into groups and given 5 mg/kg rhTRAIL, 20 mg/kg sunitinib, or the combination once daily for 5 days per week for 3 consecutive weeks. Treatment with either rhTRAIL or sunitinib alone resulted in a significant decrease in bioluminescence in the HCT116-luc tumor model (Figure 5A). Similar results were observed when mean tumor volume was measured in both the HCT116 and HCT15 tumor models compared to vehicle treated controls (Figure 5B). Consistent with our in vitro results, sunitinib significantly enhanced the efficacy of rhTRAIL in both HCT116 and HCT15 models (Figure 5A,B). In addition, all drug treatments were very well tolerated, as no significant animal weight loss was observed in either xenograft study (Figure 5C).

### 2.5. The Combination of Sunitinib and rhTRAIL Significantly Enhances JNK Phosphorylation, Reduces XIAP Levels, and Induces Apoptosis In Vivo

**Figure 6:**
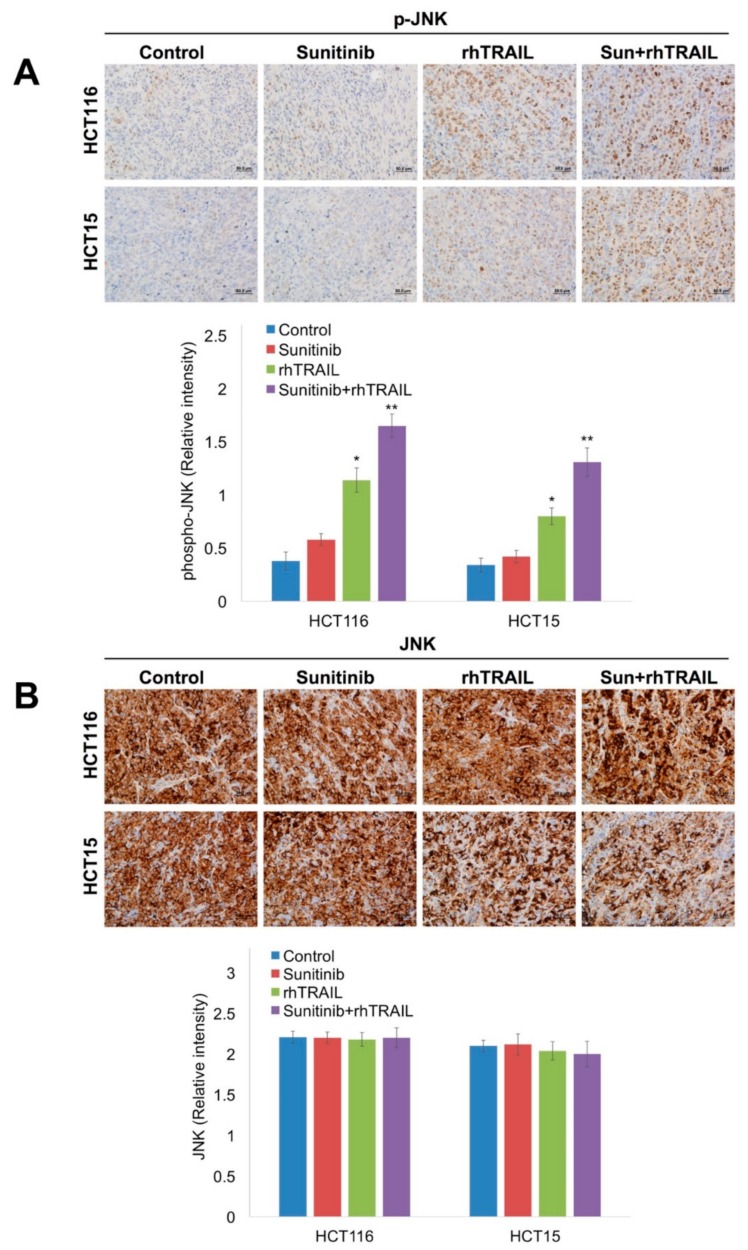
The combination of sunitinib and recombinant human tumor necrosis factor-related apoptosis-inducing ligand (rhTRAIL) significantly induces c-Jun N-terminal kinase (JNK) phosphorylation. (**A**,**B**) Slides cut from formalin-fixed paraffin-embedded tissues were deparaffinized in xylene and rehydrated. Tumors were stained with an (A) anti-p-JNK or (B) anti-JNK antibody at 4 °C overnight. Tumors were then incubated for 1 h at room temperature with goat anti-rabbit horseradish peroxidase (HRP) antibody, followed by 3,3′-diaminobenzidine for 10 min. Slides were counterstained with hematoxylin. Quantification of expression levels of both p-JNK and JNK in tumor tissue is shown. Mean ± Standard Deviation (SD), *n* = 5. * Indicates a significant difference compared to vehicle control or ** compared to either single agent group, *p* < 0.05.

**Figure 7:**
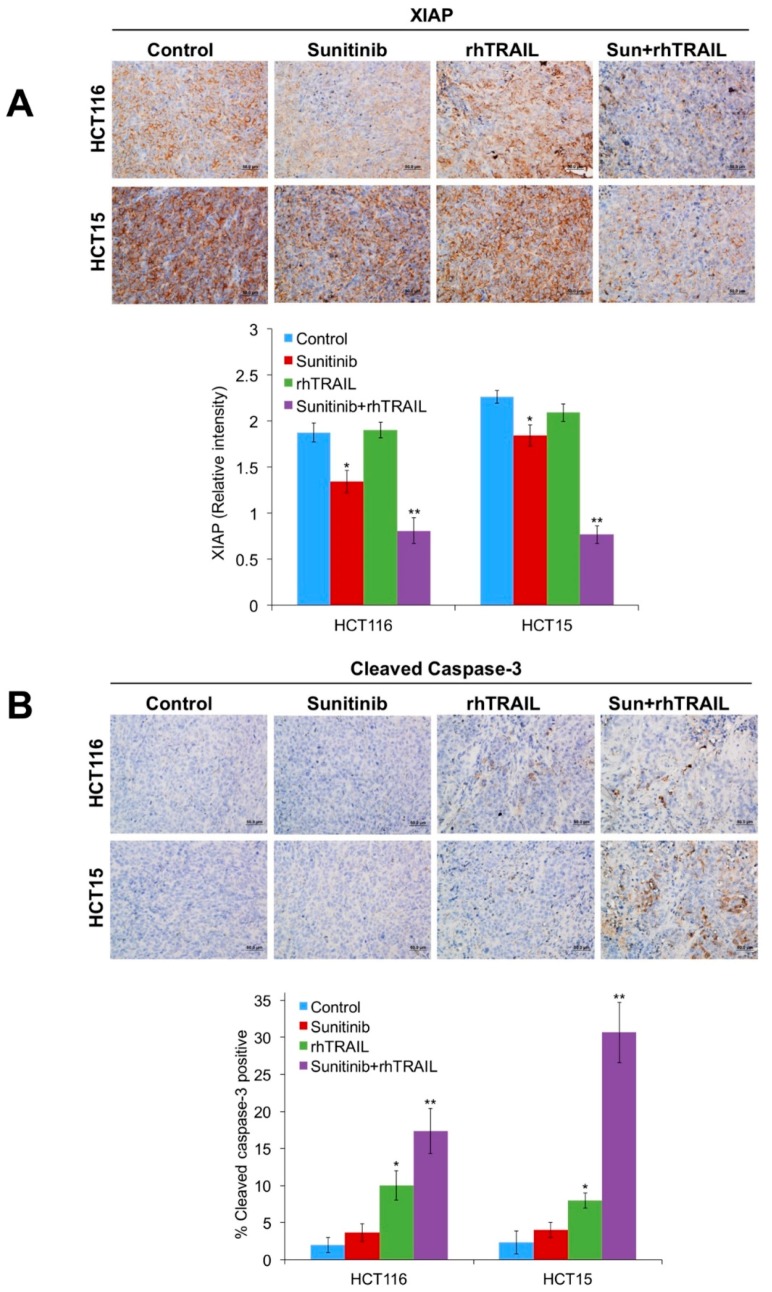
The combination of sunitinib and recombinant human tumor necrosis factor-related apoptosis-inducing ligand (rhTRAIL) significantly decreases X-linked inhibitor of apoptosis protein (XIAP) expression and increases cleaved caspase-3 levels. (**A**,**B**) Slides were stained with an (A) anti-XIAP or (B) anti-cleaved caspase-3 antibody at 4 °C overnight. Tumors were then incubated for 1 h at room temperature with goat anti-rabbit horseradish peroxidase (HRP) antibody, followed by 3,3′-diaminobenzidine for 10 min. Slides were counterstained with hematoxylin. Quantification of expression levels of both XIAP and cleaved caspase-3-positive cells in tumor tissue is shown. Mean ± Standard Deviation (SD), *n* = 5. * Indicates a significant difference compared to vehicle control or ** compared to either single agent group, *p* < 0.05.

Immunohistochemical analysis was performed to determine the in vivo effects of the rhTRAIL/sunitinib combination on JNK phosphorylation, XIAP, and cleaved caspase-3 levels. Paraffin-embedded tumor sections were stained with phospho-JNK and total JNK antibodies. Treatment with rhTRAIL significantly upregulated phospho-JNK expression, which was further enhanced by the addition of sunitinib (Figure 6A). The staining intensity of JNK remained consistent in the different treatment groups (Figure 6B). We next evaluated the expression levels of XIAP. While treatment with rhTRAIL showed no significant decrease in XIAP levels, sunitinib resulted in a modest decrease of XIAP-positive cells, which was significantly further reduced following combination therapy (Figure 7A). The reduction in XIAP levels was linked to significantly increased cleaved caspase-3 levels in the combination treatment groups compared to either monotherapy (Figure 7B). Taken together, our data demonstrate that concomitant enhanced JNK activation and reductions in XIAP drive the antitumor activity of the rhTRAIL/sunitinib combination.

## 3. Discussion

Many formulations of death receptor agonists have advanced into clinical trials, but to date single agent activity for these agents has been relatively modest [28,29,30,31,32,33,34,35]. In addition, significant liver toxicity has contributed to concerns about further development of TRAIL and TRAIL-based approaches in the clinic [36,37]. However, it is possible that reformulation of TRAIL therapeutics could improve its antitumor efficacy and decrease potential off-target effects [38,39,40]. Despite the lack of prevalent major responses in the clinic, the use of TRAIL and other death receptor agonists remains a very appealing anticancer approach due to their relatively high selectivity for malignant cells [41]. There are numerous factors that likely underlie the limited clinical activity that has been observed with death receptor monotherapies. A key factor that may diminish their efficacy is the high basal expression of anti-apoptotic proteins in tumors. Elevated levels of potent anti-apoptotic factors would logically restrict the efficacy of agents such as TRAIL [6]. Overexpression of one or more anti-apoptotic proteins such BCL-2 or BCL-XL has been reported to confer resistance to TRAIL across various tumors [42,43,44,45,46]. In addition, increased expression of FLIP has been found to be a key determinant of TRAIL-mediated apoptosis in some models [47,48]. Similarly, the IAP family members cIAP1, cIAP2, or XIAP interact with caspases to block the apoptotic cascade, with XIAP being the most potent inhibitor [49,50].

Here we show that targeted downregulation of XIAP is an important driving event in TRAIL-mediated apoptosis in colon cancer models. Our findings are in agreement with prior reports that demonstrate that XIAP blocks apoptosis induced by TRAIL in other models [51,52]. We focused on the role of IAP proteins in this study due to the potent decrease of XIAP following rhTRAIL/sunitinib combination treatment. Since XIAP can inhibit apoptosis by interacting with caspase-3, it is a key target to enhance a number of modalities of pro-apoptotic cancer therapy. Consistent with this idea, tool compound XIAP inhibitors have been shown to augment TRAIL-mediated apoptosis in multiple cancer models [53,54]. Despite the preclinical success of XIAP inhibitors, they have not been further translated into clinical benefit. Therefore, we focused on the approved anticancer receptor tyrosine kinase (RTK) inhibitor sunitinib to establish the rationale for a clinically actionable strategy to improve TRAIL therapy through XIAP targeting.

A previous report demonstrated that SW620 colon cancer tumors were sensitive to TRAIL and sunitinib-mediated apoptosis. They identified several potential targets including MCL-1, XIAP, and FLIP, but did not mechanistically target these genes to demonstrate their specific roles in apoptosis [19]. In agreement with this study, we also show enhanced antitumor activity with TRAIL and sunitinib, but demonstrate that a strong decrease in XIAP levels is a major contributor to the benefit of this combination. An important cell-specific issue with the use of death receptor agonists is whether mitochondrial apoptotic cascades need to be engaged downstream to ultimately elicit cell death. In some cancer types, mitochondrial amplification is required for TRAIL-induced cell death, whereas in others the extrinsic apoptotic pathway is sufficient for TRAIL-mediated cell death [46,55]. We show that in HCT116 cancer cells, mitochondrial amplification is required for TRAIL-induced cell death since stable overexpression of BCL-2 reduced TRAIL-induced cell death. This distinction may be an essential consideration when developing strategies to effectively use death receptor agonists in the clinical setting. In cell types where mitochondrial engagement is necessary, combination with other agents that strongly activate the mitochondrial apoptotic cascade may be required to yield meaningful clinical benefit.

Activation of stress signaling proteins such as JNK has been reported following stimulation of various TNF receptor superfamily members including TNF-R1, FAS, DR4 and DR5 [24,56,57]. The role of JNK activation in apoptosis may be context dependent as it has been convincingly shown to display pro- and anti–apoptotic functions [10,58]. Our data supports a pro-apoptotic function for JNK signaling in colon cancer cells as genetic inhibition significantly limits sensitivity to rhTRAIL and sunitinib treatment. Consistent with a pro-apoptotic mechanism of JNK, a prior study identified that TRAIL-mediated JNK activation resulted in BIM phosphorylation and regulation of FAS-mediated apoptosis [23]. While we did not investigate BIM phosphorylation downstream of TRAIL-induced JNK activation in our study, it is possible that BIM is a secondary effector that connects JNK activation with cleavage of caspases and inhibition of XIAP to regulate apoptosis induced by the TRAIL and sunitinib combination. In our models, JNK phosphorylation and caspase activation appear to regulate the reduction in XIAP levels. The precise mechanism driving this effect is unknown, but it has been suggested that JNK-mediated caspase activation may enhance the cleavage of XIAP and disrupt its function [27]. Our data supports this hypothesis as co-treatment with z-vad(OMe)-fmk blocked XIAP degradation and apoptosis induced by the rhTRAIL and sunitinib combination. However, it is also possible that JNK may work through Smac/DIABLO to increase XIAP degradation [8]. To date, there are limited studies evaluating the role of JNK activation in enhancing the ability of TRAIL to augment the efficacy of RTKs. Our current findings provide one of the first reports demonstrating that sunitinib can be used as a precision agent to potentiate the anti-cancer effects of rhTRAIL in colon carcinoma through a JNK-mediated mechanism that is specifically linked to XIAP antagonism.

In summary, our study supports renewed enthusiasm for the investigation of rhTRAIL for the treatment of colon cancer. Our findings indicate that the modest cell death observed with single agent rhTRAIL in earlier clinical trials may have resulted from an inadequate consideration for potential resistance/efficacy limiting mechanisms that could be potentially ablated through rational combination approaches. Indeed, we demonstrate here that combination therapy of rhTRAIL and sunitinib results in significantly more antitumor activity in both in vitro and in vivo colon cancer models. Importantly, adverse events/toxicity associated with sunitinib treatment are manageable and significant hepatoxicity has not been observed in patients receiving this agent [59,60]. Therefore, no overlapping toxicities are expected with combined rhTRAIL and sunitinib treatment. Mechanistically, we observed that pro-apoptotic activation of JNK following rhTRAIL treatment was further enhanced with sunitinib. JNK-related reductions in XIAP levels were directly linked to the benefit of this combination strategy. Our findings provide strong rationale for the clinical evaluation of sunitinib and other JNK activating/XIAP reducing agents in combination with death receptor agonists.

## 4. Materials and Methods 

### 4.1. Cells and Cell Culture

The human colon carcinoma cell lines, HCT116 and HCT15 were obtained from the American Type Culture Collection (Manassas, VA, USA). HCT116-luc cells were purchased from PerkinElmer (Waltham, MA, USA). The cell lines were maintained in RPMI media supplemented with 50 U/mL penicillin and 50 mg/mL streptomycin, 2 mM Glutamine, and 10% fetal bovine serum in a humidified incubator at 37 °C with 5% CO_2_. All cell lines were routinely tested for mycoplasma contamination to ensure that only negative cells were used.

### 4.2. Antibodies and Reagents

Antibodies were obtained from the following commercial sources: p-JNK (#4668), JNK (#9252), caspase-8 (#9746), BCL-2 (#4223), c-IAP-1 (#4952), c-IAP-2 (#3130), survivin (#2808), cleaved caspase-3 (#9661), FLIP (#56343), and XIAP (#2045) (Cell Signaling Technology, Danvers, MA, USA), β–Actin (sc-58673) (Santa Cruz Technologies, Santa Cruz, CA, USA), and tubulin (T7816) (Sigma, St. Louis, MO, USA). For detection, appropriate goat anti-rabbit and goat anti-rat horseradish peroxidase (HRP)-conjugated secondary antibodies (Jackson Laboratories, West Grove, PA, USA), rat anti-mouse IgG2a-HRP (Serotec, Raleigh, NC, USA), and sheep anti-mouse-HRP and donkey anti-rabbit-HRP (Amersham, Pittsburgh, PA, USA) were used. Sunitinib was purchased from the hospital pharmacy. To induce apoptosis, cells were treated with rhTRAIL (non-tagged, fragment of amino acids 114-281, Triskel Therapeutics, Groningen, The Netherlands).

### 4.3. Quantification of Drug-Induced Cytotoxicity

Cell viability was assessed by 3-(4,5-dimethylthiazol-2-yl)-2,5-diphenyltetrazolium bromide (MTT) assay. Cells were seeded into 96-well microculture plates at 10,000 cells per well and allowed to attach for 24 h. Cells were then treated with sunitinib and increasing concentrations of rhTRAIL or the combination for 72 h. Following drug treatment, MTT was added and cell viability was quantified using a BioTek (BioTek Instruments, Inc., Winooski, VT, USA) microplate reader. DNA fragmentation following in vitro drug exposure was quantified by propidium iodide (PI) staining and fluorescence-activated cell sorting (FACS) analysis. Briefly, cells were treated with rhTRAIL, sunitinib, and the combination for 24 h. Cells were then harvested and incubated with PI solution (50 μg/mL PI, 0.1% Triton-X-100, and 0.1% sodium citrate in phosphate buffered saline (PBS). Flow cytometric analysis was used to quantify cells with subdiploid DNA as a measure of apoptosis.

### 4.4. Immunoblotting

HCT116 cells were treated with 100ng/mL rhTRAIL alone or in combination with sunitinib (5 µM). Samples were prepared after indicated time intervals. Cells were collected using a cell scraper at 4 °C and were then lysed as previously described [61]. Approximately 50 μg of total cellular protein from each sample were subjected to sodium dodecyl sulfate-polyacrylamide gel electrophoresis (SDS-PAGE). Proteins were then transferred to nitrocellulose membranes and blocked with 5% nonfat milk in a Tris-buffered saline solution containing 0.1% Tween-20 for 1 h. The blots were then probed overnight with the relevant primary antibodies, washed, and probed with species-specific secondary antibodies coupled to HRP. Immunoreactive material was detected by enhanced chemiluminescence (West Pico, Pierce, Inc., Rockville, IL, USA).

### 4.5. Preparation and Transfection of shRNAs

HCT116 cells grown in 24-well plates were transfected with lentiviral shRNA particles (Santa Cruz Biotech, Santa Cruz, CA, USA) targeting JNK, FLIP, XIAP, or non-targeted (Control) in the presence of polybrene (Santa Cruz Biotech, Santa Cruz, CA, USA). Cells were cultured in puromycin to select positive cells. Efficiency of RNAi was measured by immunoblotting. HCT116 cells that were stably transfected with control vector (GFP) or BCL-2 were previously described [55].

### 4.6. Xenograft Studies

HCT116-luc and HCT15 colon cancer cancer cells (1 × 10^7^) were harvested, washed in PBS, and suspended in a mixture of Hanks’ balanced salt soultion (HBSS) and Matrigel (BD BioSciences, San Jose, CA, USA). Cells were then subcutaneously implanted into female nude mice (BALB/c background) from Harlan (Indianapolis, IN, USA). Tumor-bearing animals were randomized into treatment groups. Mice were treated with vehicle, sunitinib (20 mg/kg per os, PO), rhTRAIL (5 mg/kg intraperitoneal, IP), or both agents daily for 5 days a week for 3 weeks. Mice were monitored daily and tumor volumes were measured twice per week. To monitor the tumor growth noninvasively, HCT116-luc cells were transduced with a luciferase gene from the *Photinus pyralis* firefly. The animals were imaged using a Xenogen imaging system (Caliper Life Sciences, Hopkinton, MA, USA). After the treatment period (Day 22), HCT116-luc tumor–bearing mice received 180 mg/kg luciferin IP and were imaged 10 min after luciferin injection. At the completion of the study, tumors were excised, formalin-fixed and paraffin-embedded for immunohistochemical analysis. All animal studies were conducted in compliance with our protocol (#16-094) for animal care and use, which was approved by the Institutional Animal Care and Use Committee (IACUC) on 6 April 2016 at the University of Arizona.

### 4.7. Immunohistochemistry

Immunohistochemistry studies were conducted as previously described [62]. Briefly, paraffin-embedded tumor sections (5 µm thick) were mounted on slides. Sections were deparaffinized in xylene, treated with a graded series of alcohol, and rehydrated in PBS (pH 7.5). Heat-induced epitope retrieval was performed by microwaving slides in a citrate buffer for 5 min. The slides were allowed to cool and endogenous peroxides were blocked with a 3% hydrogen peroxide solution for 10 min. Slides were then incubated in a protein block solution (5% horse and 1% goat serum in PBS) for 20 min. Primary antibodies (phospho-JNK, ab4821, JNK, ab112501, abcam, Cambridge, MA, USA); (XIAP, #14334, cleaved caspase-3, #9661, Cell Signaling, Danvers, MA, USA) were diluted in the protein block solution and placed at 4 °C overnight. After washing with PBS, slides were incubated in appropriate secondary antibodies for 1 h at ambient temperature. Positive reactions were visualized by immersing the slides with stable 3,3′-diaminobenzidine diaminobenzidine (Research Genetics, Huntsville, AL, USA) for 10 min. The sections were rinsed with distilled water, counterstained with Gill’s hematoxylin (Sigma, St. Louis, MO, USA), and mounted with Universal Mount (Research Genetics, Huntsville, AL, USA). Images were captured using an Olympus fluorescent microscope (Center Valley, PA, USA) with a DP71 camera and a 20x objective. Image-Pro Plus software Version 6.2.1 (MediaCybernetics, Bethesda, MD, USA) was used for image acquisition and quantification by densitometric analysis (p-JNK and JNK) of five random high-power fields containing viable tumor cells. Quantification of proliferating cell nuclear antigen (PCNA) and cleaved caspase-3 was conducted by counting the number of positive cells in five random fields.

### 4.8. Statistical Analyses

Statistical significance of differences observed between samples was determined using the Tukey–Kramer comparison test or the Student’s t test. Differences were considered significant in all experiments at *p* < 0.05.

## 5. Conclusions

Treatment with the approved agent sunitinib significantly improved the anticancer activity of TRAIL by inducing enhanced JNK activation, which resulted in a strong reduction in XIAP levels. Since XIAP is the most potent inhibitor of apoptosis of the IAP proteins, targeting its expression is a promising approach to augment the efficacy of pro-apoptotic agents, such as TRAIL. Given the ability of the rhTRAIL/sunitinib combination to decrease XIAP levels in both in vitro and in vivo colon cancer models, additional studies are warranted to evaluate this therapeutic approach.
